# Revisiting regulation of potassium homeostasis in *Escherichia coli*: the connection to phosphate limitation

**DOI:** 10.1002/mbo3.438

**Published:** 2017-01-17

**Authors:** Hannah Schramke, Vera Laermann, Halina E. Tegetmeyer, Andreas Brachmann, Kirsten Jung, Karlheinz Altendorf

**Affiliations:** ^1^ Department of Biology I, MicrobiologyCenter for integrated Protein Science Munich (CiPSM)Ludwig‐Maximilians‐Universität MünchenMartinsriedGermany; ^2^ Fachbereich Biologie/ChemieUniversität OsnabrückOsnabrückGermany; ^3^ Centrum für BiotechnologieUniversität BielefeldBielefeldGermany; ^4^ Alfred Wegener InstituteHelmholtz Centre for Polar and Marine ResearchBremerhavenGermany; ^5^ Max Planck Institute for Marine MicrobiologyBremenGermany; ^6^ Department of Biology I, GeneticsLudwig‐Maximilians‐Universität MünchenMartinsriedGermany; ^7^ Present address: Molecular Systems BiologyGroningen Biomolecular Sciences and Biotechnology InstituteUniversity of GroningenGroningenThe Netherlands

**Keywords:** cross‐regulation, histidine kinase, ion homeostasis, response regulator, signal transduction

## Abstract

Two‐component signal transduction constitutes the predominant strategy used by bacteria to adapt to fluctuating environments. The KdpD/KdpE system is one of the most widespread, and is crucial for K^+^ homeostasis. In *Escherichia coli*, the histidine kinase KdpD senses K^+^ availability, whereas the response regulator KdpE activates synthesis of the high‐affinity K^+^ uptake system KdpFABC. Here we show that, in the absence of KdpD, *kdpFABC* expression can be activated via phosphorylation of KdpE by the histidine kinase PhoR. PhoR and its cognate response regulator PhoB comprise a phosphate‐responsive two‐component system, which senses phosphate limitation indirectly through the phosphate transporter PstCAB and its accessory protein PhoU. In vivo two‐hybrid interaction studies based on the bacterial adenylate cyclase reveal pairwise interactions between KdpD, PhoR, and PhoU. Finally, we demonstrate that cross‐regulation between the *kdpFABC* and *pstSCAB* operons occurs in both directions under simultaneous K^+^ and phosphate limitation, both in vitro and in vivo. This study for the first time demonstrates direct coupling between intracellular K^+^ and phosphate homeostasis and provides a mechanism for fine‐tuning of the balance between positively and negatively charged ions in the bacterial cell.

## 1 INTRODUCTION

Potassium ion (K^+^) is the most abundant cation in cells and is required for several cellular processes, including maintenance of turgor (Epstein, 2003), regulation of intracellular pH (Booth, 1985) and activation of enzymes (Nissen, Hansen, Ban, Moore, & Steitz, 2000). *Escherichia coli* maintains high intracellular K^+^ concentrations (200–400 mmol/L) by uptake via the low‐affinity transporters Trk and Kup and the high‐affinity transporter KdpFABC. Synthesis of KdpFABC is in turn up‐regulated by the KdpD/KdpE two‐component system in response to K^+^ limitation and osmotic stress. Under K^+^ limitation ([K^+^]_ex_ <5 mmol/L) the histidine kinase KdpD autophosphorylates and transfers the phosphoryl group to the response regulator KdpE. The phosphorylated form of KdpE dimerizes and thereby activates transcription of *kdpFABC* (Sugiura, Nakashima, Tanaka, & Mizuno, 1992; Voelkner, Puppe, & Altendorf, 1993). The counteracting phosphatase activity of KdpD is responsible for dephosphorylation of KdpE‐P in the presence of K^+^ and thereby terminates *kdpFABC* expression (Jung, Tjaden, & Altendorf, 1997). However, the nature of the stimulus sensed by KdpD is a long‐standing puzzle. The protein was initially thought to respond to a decrease in turgor or some effect thereof (Laimins, Rhoads, & Epstein, 1981; Malli & Epstein, 1998). However, measurements of cytoplasmic volumes after exposure to various osmolytes disproved this hypothesis (Hamann, Zimmann, & Altendorf, 2008). The current view is that KdpD directly perceives three chemical stimuli: intracellular (Heermann et al., 2014; Schramke, Tostevin, Heermann, Gerland, & Jung, 2016) and extracellular (Laermann, Cudic, Kipschull, Zimmann, & Altendorf, 2013; Schramke et al., 2016) K^+^ concentrations, ionic strength (Jung, Veen, & Altendorf, 2000) and ATP levels (Heermann, Altendorf, & Jung, 2000; Jung & Altendorf, 1998b). In addition to responding to these chemical stimuli, KdpD activity is influenced by accessory proteins. Under conditions of osmotic stress, the universal stress protein UspC acts as a scaffold for the KdpD/KdpE signaling cascade (Heermann et al., 2009) and therefore circumvents the inhibitory effect of K^+^ under these conditions. Furthermore, it has been shown that dephosphorylated enzyme IIA^Ntr^, which is part of the Ntr phosphotransferase system, can bind KdpD, modulates its activity and therefore links carbohydrate metabolism to K^+^ homeostasis (Lüttmann et al., 2009).

It has long been known that K^+^ is required for phosphate $\left({\text{PO}}_{4}^{3-}\right)$ uptake (Weiden, Epstein, & Schultz, 1967). Phosphate is crucial for the synthesis of cellular components such as membrane lipids or nucleic acids, as well as for signal transduction processes (Crépin et al., 2011; Santos‐Beneit, 2015). Free intracellular ${\text{PO}}_{4}^{3-}$ levels in *E. coli* range from 5 to 20 mmol/L, depending on the growth rate and carbon source (Rao, Roberts, Torriani, & Yashphe, 1993; Shulman et al., 1979; Ugurbil, Rottenberg, Glynn, & Shulman, 1978, 1982; Xavier, Kossmann, Santos, & Boos, 1995). Upon phosphate limitation ([${\text{PO}}_{4}^{3-}$]_ex_ <4 μmol/L), the two‐component system PhoR/PhoB regulates expression of more than 30 genes belonging to the *pho* regulon (Hsieh & Wanner, 2010). The *pho* regulon comprises genes coding for proteins that are important for phosphate assimilation (Hsieh & Wanner, 2010) and the timing of their expression, as well as their production levels, are determined by the binding affinity of PhoB for the corresponding promoters (Gao & Stock, 2015). One of the targets of PhoB is the *pst* operon, which encodes the high‐affinity phosphate transporter Pst (*K*_*m*_^app^ ≈0.2 μmol/L (Rosenberg, Gerdes, & Chegwidden, 1977; Willsky & Malamy, 1980)). The Pst transporter belongs to the ATP‐binding cassette (ABC) family of transporters and comprises the periplasmic phosphate‐binding protein PstS, the two transmembrane channel‐forming factors PstA and PstC and the ATPase PstB. It is rather unclear how the histidine kinase PhoR senses the availability of ${\text{PO}}_{4}^{3-}$. It is suggested that PhoR monitors the activity of the high‐affinity ${\text{PO}}_{4}^{3-}$ transporter PstCAB via PhoU, which interacts with both PstB and PhoR and thereby probably modulates PhoR activity (Gardner, Johns, Tanner, & McCleary, 2014). However, PhoU is not involved in phosphate sensing in *Caulobacter crescentus* (Lubin, Henry, Fiebig, Crosson, & Laub, 2016). In *E. coli* PstCAB and PhoU have an inhibitory effect on PhoR, as the absence of any one of these components results in constitutive expression of the *pho* regulon (Hsieh & Wanner, 2010; Lamarche, Wanner, Crepin, & Harel, 2008). Moreover, it is also known that, besides PhoR, some noncognate histidine kinases such as ArcB, CreC, KdpD, QseC, EnvZ, and BaeS can stochastically activate PhoB (Zhou, Grégori, Blackman, Robinson, & Wanner, 2005).

In this study, we demonstrate that the histidine kinase PhoR can activate *kdpFABC* expression independently of KdpD, but requires functionally active KdpE to do so. Furthermore, the deletion of *phoU*—which is supposed to be a negative regulator of the PhoR/PhoB system in *E. coli*—resulted in high *kdpFABC* expression in a reporter strain lacking KdpD. Using the bacterial two‐hybrid (BACTH) system we show here that both the KdpD/KdpE and the PhoR/PhoB two‐component systems interact with PhoU in vivo. Ultimately, we find that cross‐regulation between these two systems is not just a nonphysiological curiosity, but also occurs in the presence of the partner histidine kinase under conditions of K^+^ and phosphate limitation.

## 2 EXPERIMENTAL PROCEDURES

### 2.1 Strains, plasmids, and oligonucleotides

Strains, plasmids, and oligonucleotides used in this study are listed in Tables 1, 2, 3. The strains LB2240*ΔkdpD* and LB2240Δ*kdpD,kdpE* ^*D52N*^ were constructed in two steps, using Red^®^/ET^®^ recombination technology in combination with *rpsL* counterselection (Heermann, Zeppenfeld, & Jung, 2008). Briefly, in the first step, a linear DNA fragment encoding a kanamycin cassette (amplified with the primer pairs 50bp*kdpD*_rpsL‐ kan_sense + 50bp*kdpD*_rpsL‐ kan_antisense and 50bp*kdpE*_rpsL‐ kan sense + 50bp*kdpE*_rpsL/kan_antisense; see Table 3) was inserted into the *kdpD* (for LB2240*ΔkdpD*) and *kdpE* (for LB2240*ΔkdpD,kdpE* ^*D52N*^) genes, respectively. In the second step, the kanamycin cassette was replaced by a DNA fragment encoding either the *kdpD* deletion, or the *kdpE* ^*D52N*^ substitution, respectively. The DNA fragment incorporating the *kdpD* deletion was generated by a two‐step PCR using genomic DNA of LB2240 as template and the primer pairs kdpCDforI_sense + Δ*kdpD*_antisense and Δ*kdpD*_sense + kdpE_antisense (Table 3). The fragment bearing the *kdpE* ^*D52N*^ substitution was derived from pPV‐2/D52N by amplification with the primer pairs *kdpE*_sense +* kdpE*_antisense (Table 3). LB2240*ΔkdpDΔptaΔackA* and LF3*ΔkdpD* were constructed using Quick and Easy *E. coli* Gene Deletion and Bac Modification Kits (Gene Bridges) as previously described (Heermann et al., 2008). Briefly, we inserted a linear DNA fragment encoding a kanamycin cassette (obtained by amplification with the primer pairs 50bp*ackApta*_rpsL‐kan_sense + 50bp*ackApta*_rpsL‐kan_antisense or delta KdpD_up + delta KdpD_down; Table 3) as selection marker into the genes *pta, ackA* (parental strain LB2240*ΔkdpD*) and *kdpD* (parental strain LF3)*,* respectively. To avoid effects of the kanamycin resistance cassette on *kdpE* expression levels, we removed the selection marker in LF3*ΔkdpD* using the pCP20 helper plasmid as described previously (Baba et al., 2006). LB2240*ΔpstC*, LB2240*ΔkdpDΔpstC* and all other LF3 deletion mutants (Table 1) were constructed by P1 transduction (Miller, 1972). Strains JW3705 (*pstC::npt*), JW0390 (*phoR::npt*) and JW3702 (*phoU::npt*) were used as donor strains (Baba et al., 2006). Preparation of phage lysate from donor strains and transduction to recipient strains was performed as described previously (Leder, Tiemeier, & Enquist, 1977). For double or triple deletions the kanamycin cassette was removed between steps using the helper plasmid pCP20 as previously described (Datsenko & Wanner, 2000). Successful deletion was confirmed by PCR using appropriate primers listed in Table 3.

**Table 1:** Strains used in this study

Name	Genotype	Reference
MG1655	Wild‐type K‐12 strain	(Blattner et al., 1997)
BL21 (DE3/pLysS)	*F* ^*‐*^ *ompT hsdSB (r* ^*−*^_*B*_ *m* ^*−*^_*B*_ *) dcm gal (DE3) pLysS* (Cm^R^)	(Studier & Moffatt, 1986)
TKR2000	*∆kdpFABCDE trkA405 trkD1 atp706*	(Kollmann & Altendorf, 1993)
LB2240	*thi metE rpsL gal rha kup1 ΔtrkA*	P1 (TK2240) → LB2003; this work
TK2240	*thi rha lacZ nagA kup1 trkA405*	(Epstein, Whitelaw, & Hesse, 1978)
LB2003	*thi metE rpsL gal rha kup1 ΔtrkA* Δ*kdpABC5*	(Stumpe & Bakker, 1997)
TKV2209	*ΔkdpD* ^*128‐894*^ *ΔkdpE trkA405 trkD1 nagA thi rha lacZ*	(Zimmann, Puppe, & Altendorf, 1995)
BTH101	F^−^ *cyaA*‐*99 araD139 galE15 galK16 rpsL1 hsdR2 mcrA1 mcrB1*	(Karimova, Pidoux, Ullmann, & Ladant, 1998)
JW3705	BW25113 *pstC::npt*	(Baba et al., 2006)
JW0390	BW25113 *phoR::npt*	(Baba et al., 2006)
JW3702	BW25113 *phoU::npt*	(Baba et al., 2006)
LF3	MG1655 *rpsL150* P_*kdp*_ *::lacZ*	(Fried, Lassak & Jung 2012)
LB2240*ΔkdpD*	*ΔkdpD thi metE rpsL150 gal rha kup1 ΔtrkA*	This work
LB2240*ΔkdpD,kdpE* ^*D52N*^	*ΔkdpDkdpED52N thi metE rpsL150 gal rha kup1 ΔtrkA*	This work
LB2240 *ΔkdpDΔptaΔackA*	*ΔkdpD (pta ackA)::rpsL‐kan thi metE rpsL150 gal rha kup1 ΔtrkA*	This work
LB2240*ΔpstC*	LB2240 *pstC::npt*	This work
LB2240 *ΔkdpDΔpstC*	LB2240*ΔkdpD pstC::npt*	This work
LF3*ΔkdpD*	MG1655 *rpsL150* P_*kdp*_ *::lacZ ΔkdpD*	This work
LF3*ΔpstC*	LF3 *pstC::npt*	This work
LF3 *ΔkdpDΔpstC*	MG1655 *rpsL150* P_*kdp*_ *::lacZ ΔkdpD pstC::npt*	This work
LF3 *ΔkdpDΔpstCΔphoR*	MG1655 *rpsL150* P_*kdp*_ *::lacZ ΔkdpD ΔpstC phoR::npt*	This work
LF3 *ΔkdpDΔphoU*	MG1655 *rpsL150* P_*kdp*_ *::lacZ ΔkdpD phoU::npt*	This work
LF3 *ΔkdpDΔphoR*	MG1655 *rpsL150* P_*kdp*_ *::lacZ ΔkdpD phoR::npt*	This work
LF3 *ΔkdpDΔphoUΔphoR*	MG1655 *rpsL150* P_*kdp*_ *::lacZ ΔkdpD ΔphoU phoR::npt*	This work

**Table 2:** Plasmids used in this study

Name	Relevant genotype or description	Reference
pBD5‐9	*kdpD* in pBAD18	(Zimmann, Steinbrugge, Schniederberend, Jung, & Altendorf, 2007)
pRED/ET	λ‐RED recombinase in pBAD24, Ap^R^	Gene Bridges
pCP20	Helper plasmid, Cm^R^	(Datsenko & Wanner, 2000)
pPV‐2/D52N	*kdpE* ^*D52N*^ in pPV‐2	This work
pBR‐Cherry	*mcherry* in pBR322	(Münch, Stingl, Jung, & Heermann, 2008)
pPV5‐3	*kdpD6His* in pKK223‐3	(Jung & Altendorf, 1998a)
pEE	*10His‐kdpE* under *P*_*T7*_ control in pET16B*,* Amp^r^	(Heermann et al., 2003)
pDL39	*phoB*‐His_10_ under *P*_*tac*_ control in pKES170	(Lüttmann et al., 2012)
pDL40	*phoR*(codons 52‐431)‐His_10_ under *P*_*tac*_ control in pKES170	(Lüttmann et al., 2012)
pUT18	Expression vector, Ap^R^	(Karimova & Ladant, 2005)
pUT18C	Expression vector, Ap^R^	(Karimova & Ladant, 2005)
pKT25	Expression vector, Km^R^	(Karimova & Ladant, 2005)
pUT18C‐zip	Control plasmid, N‐terminal CyaA‐T18‐yeast leucine‐zipper fusion, Ap^R^	(Karimova & Ladant, 2005)
pKT25‐zip	Control plasmid, N‐terminal CyaA‐T25‐yeast leucine zipper fusion, Km^R^	(Karimova & Ladant, 2005)
pUT18‐gene	Gene of interest cloned into the PstI and BamHI sites of pUT18, resulting in C‐terminal CyaA‐T18‐protein fusions (T18‐gene)	This work
pUT18C‐gene	Gene of interest cloned into the PstI and BamHI sites of pUT18, resulting in N‐terminal CyaA‐T18‐protein fusions (gene‐T18)	This work
pKT25‐gene	Gene of interest cloned into the PstI and BamHI/EcoRI sites of pKT25, resulting in N‐terminal CyaA‐T25‐protein fusions (gene‐T25)	This work
pBR‐Cherry pPstS	P_*pstS*_ promoter region in pBR‐Cherry	This work

**Table 3:** Oligonucleotides used in this study

Name	Sequence (5′‐3′)
Primers for construction of deletion strains	
delta KdpD_up	TCTTCCAGCGTTAACCACTCTTTCTTCAAATTAAAGCCGCCGGACTGAATAATTAACCCTCACTAAAGGGCG
delta KdpD_down	ACTCAATCTGGCGCTGGATAAACTTGATGAATAACGAACCCTTACGTCCCTAATACGACTCACTATAGGGCTC
50bp*kdpD*_rpsL‐ kan_sense	CCAGCCGGTTGTCAACATTGTTGAACTCAATCTGGCGCTCGACAAACTTGACGAATAAGGCCTGGTGATGATGGCGGGATCGTTG
50bp*kdpD*_rpsL‐ kan_antisense	GCGCAGAAAGCGACGAATAGCCTGTTCATCTTCAACAATCAGAACGTTTGTCAGAAGAACTCGTCAAGAAGGCGATAG
Δ*kdpD*_sense	GACGAATAACAAACGTTCTGATTGTTGAAGATG
Δ*kdpD*_antisense	CAGAACGTTTGTTATTCGTCAAGTTTGTCGAGCGCCAGATTGAG
kdpCDforI_sense	CGCAAGCGGCGGCCTGGC
kdpE_antisense	CCGGTGAATCACGCGGGCGGC
50bp*kdpE*_rpsL‐ kan sense	CTGCGCACGGCGCTGGAGGGCGACGGGATGCGCGTCTTTGAGGCCGAAACGGCCTGGTGATGATGGCGGG
50bp*kdpE*_rpsL/kan_antisense	TTTACCAGCGGATCGGGCGCGGTGGTGGCAGAGTGGCGGCGTAATGCGACTCAGAAGAACTCGTCAAGAAG
*kdpE*_sense	CCGGTGAATCACGCGGGCGGC
*kdpE*_antisense	CCGGAGCGGATGATTATCTG
50bp*ackApta*_rpsL‐kan_sense	TAACGATAACGCCGGTGATGTTGGTGTTTTTGGCACCGCCGAAGCTGTTGGGCCTGGTGATGATGGCGGG
50bp*ackApta*_rpsL‐kan_antisense	GGCCTAAGTAGTACATATTCATTGAGTCGTCAAATTCATATACATTATGCTCAGAAGAACTCGTCAAGAAG
Check primers for deletion strains	
check kdpD_s	ATCACCGGCACCGCGCTCCACTGGCGC
check kdpD_as	GTTCCGGTTGAACTGGTGACGGCATCG
check pstC_s	CGTTCGCCAGACCACCACCTTCC
check pstC_as	CTGAAATTCTTCGACTGGGCGTAC
check phoR_s	CCGCACGGTCGATGTCCACATTC
check phoR_as	CAGTATGACAGCACCTGAAGCGC
check phoU_s	CACCGTGGTGATCGTCACCC
check phoU_as	GTTATGTCAGGTTTTGCCTGCGA
Primers for construction of BACTH plasmids	
KdpD_PstI_s_pUT18C	TTGGCTGCAGCATGAATAACGAACCCTTACG
KdpD_BamHI_as_pUT18C	GACGGATCCTCACATATCCTCATGAAATTCTTC
KdpE_PstI_s_pKT25	TTGGCTGCAGCAGTGACAAACGTTCTGATTGTTG
KdpE_EcoRI_as_pUT18C+ pKT25	GACGAATTCGTCAAAGCATAAACCGATAGCCAAT
PhoR_PstI_s_pUT18 + C	TTGGCTGCAGCGTGCTGGAACGGCTGTCGTGG
PhoR_BamHI_as_pUT18C + pKT25	GATCGGATCCTTAATCGCTGTTTTTGGC
PhoR_PstI_s_pKT25	TTGGCTGCAGCAGTGCTGGAACGGCTGTCGTGG
PhoU_PstI_s_pUT18 + C	TTGGCTGCAGCATGGACAGTCTCAATCTTAATA
PhoU_BamHI_as_pUT18	GACGGATCCGATTTGTCGCTATCTTTCCCCG
PhoU_PstI_s_pKT25	TTGGCTGCAGCAATGGACAGTCTCAATCTTAATA
PhoU_BamHI_as_pUT18C + pKT25	GATCGGATCCTTATTTGTCGCTATCTTTCC
PhoB_PstI_s_pUT18 + C	TTGGCTGCAGCATGGCGAGACGTATTCTGGTCG
PhoB_BamHI_as_pUT18	GACGGATCCGAAAAGCGGGTTGAAAAACGATAT
PhoB_PstI_s_pKT25	TTGGCTGCAGCAATGGCGAGACGTATTCTG
PhoB_BamHI_as_pUT18C+pKT25	GATCGGATCCTTTAAAAGCGGGTTGAAAAAC
qRT‐PCR primers	
KdpAfor2	GCCGCCAGCGGGATTGCGG
KdpArev2	CTTCAACGGTATTCACAGCCTG
KdpDfor	CGCCGCCATGCTGGAAGGGC
KdpDrev	GCTTCCAGCAGTTCTTCGATATC
GapAfor1	CTCCACTCACGGCCGTTTCG
GapArev1	CTTCGCACCAGCGGTGATGTG
Primers for construction of *mCherry* reporter plasmid	
pPstS_BamHI_s	GATCGGATCCTCTTCGCCGATCAGGATGCG
pPstS_XmaI_as	GATCCCCGGGAATGTCTCCTGGGAGGATTC

Plasmids for the bacterial adenylate cyclase assays (BACTH) were constructed by DNA amplification using genomic DNA of *E. coli* MG1655 as template with primer pairs listed in Table 3, and subsequent cloning into the indicated vectors. Successful insertion was confirmed by restriction analysis with appropriate enzymes.

Plasmid pBR‐Cherry pPstS was constructed by amplification of the region upstream of the *pstS* gene (~500 bp) using primers pPstS_BamHI_s and pPstS_XmaI_as (Table 3) and genomic DNA of *E. coli* MG1655 as template. After restriction with XmaI and BamHI, the DNA fragment was ligated into pBR‐Cherry. Successful cloning was confirmed by restriction and sequencing analyses.

### 2.2 Molecular biological techniques

Plasmid DNA was isolated using the HiYield Plasmid Minikit (Suedlaborbedarf) or the QIAaprep Spin Miniprep Kit (Qiagen), respectively. Genomic DNA was isolated using the DNeasy Tissue Kit (Qiagen) and the UltraClean Microbial DNA Isolation Kit (MO BIO), respectively. DNA fragments were purified from agarose gels using a HiYield PCR Cleanup and Gel Extraction Kit (Suedlaborbedarf) or the QIAquick Gel Extraction Kit (Qiagen), respectively. Q5 DNA polymerase (New England BioLabs) and OneTaq DNA polymerase (New England BioLabs) were used according to the supplier's instructions. Restriction enzymes and other DNA‐modifying enzymes were also purchased from New England BioLabs and used according to the manufacturer's directions.

### 2.3 Growth conditions

KML complex medium [1% (w/v) KCl, 1% (w/v) tryptone, 0.5% (w/v) yeast extract] was used as standard medium for strains TKR2000, LB2240 and derivatives, and Lysogeny Broth [1% (w/v) NaCl, 1% (w/v) tryptone, 0.5% (w/v) yeast extract] for MG1655, BL21 (DE3/pLysS), LF3 and derivatives, respectively. To analyze K^+^‐dependent growth and reporter gene expression we used a phosphate‐buffered minimal medium containing the indicated K^+^ concentrations (Epstein & Kim, 1971). For growth of cells on different ${\text{PO}}_{4}^{3-}$ and K^+^ concentrations we used a Tris‐maleic acid (TMA) minimal medium (Weiden et al., 1967), and KCl and Na_2_HPO_4_ were added as indicated. Glucose was added as the carbon source at a final concentration of 0.4% (w/v). Whenever necessary, thiamine was added at a final concentration of 1 μg ml^−1^. Appropriate antibiotics were added at final concentrations of 100 μg ml^−1^ (ampicillin), 50 μg ml^−1^ (kanamycin), and 25 μg ml^−1^ (chloramphenicol). For cultivation on plates 1.5% (w/v) agar was added to the corresponding medium. Unless otherwise stated, cells were grown under aeration at 37°C.

### 2.4 RNA isolation, cDNA synthesis, and qRT‐PCR

At indicated time points, cells were harvested and RNA was isolated using the RNeasy Mini Kit (Qiagen) according to the manufacturer's directions. RNA concentration was adjusted to 20 μg ml^−1^ and treated with RNAse‐free DNAse (New England Biolabs) for 60 min at 37°C to remove residual chromosomal DNA. Subsequently, DNAse was heat‐inactivated for 5 min at 70°C and RNA was stored at −20°C. cDNA was synthesized using the RevertAid first‐strand cDNA synthesis kit (Fermentas) according to the manufacturer's directions, subsequently samples were cooled to 4°C and immediately frozen at −20°C. Quantitative real‐time PCR (qRT‐PCR; iQ5 Real‐Time PCR Detection System; Bio‐Rad) was performed using primers specific for *kdpA*,* kdpD,* and *gapA* (see Table 3). The cycle threshold (C_T_) value was determined after 40 cycles using iQ software (Bio‐Rad) and values were normalized with reference to the value of *gapA*.

### 2.5 Whole‐genome shotgun sequencing

Illumina Nextera libraries were generated (as recommended in the Nextera DNA Sample Preparation Guide) from genomic DNAs extracted and purified from *E. coli* LB2240Δ*kdpD* and its mutant (LB2240ΔkdpD*) derivatives. The libraries were sequenced on a MiSeq instrument in a 2 × 300 bases paired‐end run. Sequencing depth was between 270‐ and 300‐fold. Prior to sequence analysis, the sequenced reads were quality trimmed using trimmomatic v3.0 (Bolger, Lohse, & Usadel, 2014) (settings: PE mode, headcrop 15 bases, sliding window length 3 with min. quality 20, crop trailing bases below q20, crop to length of 284 bases, discard reads shorter than 50 bases after q‐trimming). Reads of mutant and parental strains were separately assembled onto the *E. coli* K12 MG1655 reference genome.

### 2.6 Genome sequence analysis

Quality‐trimmed sequence reads were aligned to the most closely related published genome, *E. coli* K12 MG1655 (GenBank Accession No. CP009685) using NovoAlign (NovoCraft Technologies) and CLC Genomics Server 7.5 (Qiagen). Alignment depth was between 270 and 290. Less than 0.15% of the reads could not be aligned. The alignments were screened for differences between the sequenced *E. coli* LB2440 mutants and the MG1655 genome according to a previously described procedure for local realignment and SNP and indel detection (Dettman et al., 2012), adjusting the settings to suit the analyzed data. Finally, alignments were manually examined for differences between LB2240*ΔkdpD* and LB2240ΔkdpD* strains using the samtools pileup output (Dettman et al., 2012) and ReadXplorer (Hilker et al., 2014) for alignment visualization.

The LB2240 mutant sequence reads are publicly available in GenBank under the BioProject PRJNA322678.

### 2.7 β‐Galactosidase activity assays (determination of *kdpFABC* expression in vivo)

In vivo *kdpFABC* expression was analyzed using strains LF3 and derivatives (P_*kdpFABC*_ *::lacZ*) thereof (Table 1). Cells were aerobically grown at 37°C in minimal media containing the indicated K^+^ concentrations (Epstein & Kim, 1971; Weiden et al., 1967) and harvested by centrifugation in late exponential phase. β‐Galactosidase activity was determined as described (Miller, 1992) and is given in Miller Units.

### 2.8 P_*pstS*_ promoter activity assays

*E. coli* MG1655 carrying plasmid pBR‐Cherry pPstS was cultivated in TMA medium supplemented with the indicated KCl and Na_2_HPO_4_ concentrations. Cells were grown aerobically in a 96‐well plate in a final volume of 150 μl at 37°C. Optical density (wavelength 600 nm) and fluorescence (excitation wavelength 560 nm, emission wavelength 612 nm) were measured with a Tecan Infinite F500 system. Promoter activity was calculated as described previously (Bren, Hart, Dekel, Koster, & Alon, 2013).

### 2.9 Bacterial adenylate cyclase two‐hybrid assay (BACTH)

Protein‐protein interactions were assayed with the bacterial adenylate cyclase based two‐hybrid system (BACTH) essentially as described previously (Karimova & Ladant, 2005). *E. coli* BTH101 was transformed with different pUT18, pUT18C, and pKT25 derivatives (Table 2) to test for interactions. We used pUT18C and pKT25 as the negative control and the leucine zipper fusion constructs as positive controls, respectively (Table 2). Cells were grown under aeration for 48 hr in LB medium supplemented with the appropriate antibiotics and 0.5 mmol/L IPTG at 25°C. Subsequently, cells were harvested for determination of β‐galactosidase activities (Miller, 1992). For BATCH assays on plates, 1.5% (w/v) agar was added to the indicated medium, which in addition contained, 0.5 mmol/L isopropyl‐β‐D‐thiogalactopyranoside (IPTG), 100 μg ml^−1^ ampicillin, 50 μg ml^−1^ kanamycin, and 40 μg ml^−1^ 5‐bromo‐4‐chloro‐3‐indolyl‐β‐D‐galactopyranoside (X‐Gal). After washing cells from overnight cultures with TMA medium (without KCl or Na_2_HPO_4_), equal cell numbers were spotted on plates and incubated at 25°C for 72 hr.

### 2.10 Cell fractionation and preparation of membrane vesicles

*E. coli* strain TKR2000 transformed with plasmid pPV5‐3 was grown aerobically at 37°C to until OD_600_=1 in KML complex medium supplemented with ampicillin (100 μg ml^−1^). After harvesting, cells were washed with buffer (50 mmol/L Tris/HCl pH 7.5, 10 mmol/L MgCl_2_) and disrupted by passage through a Cell Disruptor (Constant Cell Disruption Systems, Daventry, UK) at 1.35 kbar and 4°C in disruption buffer [50 mmol/L Tris/HCl pH 7.5, 10% (v/v) glycerol, 10 mmol/L MgCl_2_, 1 mmol/L dithiothreitol, 0.5 mmol/L phenylmethylsulfonylfluoride, and 0.03 mg ml^−1^ DNase]. After removal of intact cells and cell debris by centrifugation (9,000*g*, 10 min), membrane vesicles were collected by centrifugation at 160,000*g* for 60 min. The vesicles were washed with low‐ionic‐strength buffer (10 mmol/L Tris/HCl, pH 7.5, 3 mmol/L EDTA), centrifuged again and resuspended in 50 mmol/L Tris/HCl, pH 7.5 containing 10% (v/v) glycerol. Vesicles were frozen in liquid nitrogen and stored at −80°C until use.

### 2.11 Overproduction and purification of soluble proteins

*E. coli* strain BL21 (DE3/pLysS) transformed with pDL39, pDL40 or pEE was grown aerobically at 37°C in lysogenic broth supplemented with ampicillin (100 μg ml^−1^). Gene expression was induced at OD_600_=0.5 with 0.5 mmol/L IPTG and cells transformed with pEE were grown for another 3 hr, whereas cells carrying pDL39 or pDL40 were cultivated overnight at 16°C after induction. After harvesting, cells were washed with buffer (50 mmol/L Tris/HCl pH 7.5, 10 mmol/L MgCl_2_) and disrupted by passage through a Cell Disruptor (Constant Cell Disruption Systems) at 1.35 kbar and 4°C in disruption buffer [50 mmol/L Tris/HCl pH 7.5, 10% (v/v) glycerol, 10 mmol/L MgCl_2_, 1 mmol/L dithiothreitol, 0.5 mmol/L phenylmethylsulfonylfluoride, and 0.03 mg ml^−1^ DNase]. After removal of intact cells and cell debris by centrifugation (9,000*g*, 10 min), the cytosol was frozen at −80°C. Despite the fact that the truncated version of PhoR lacks the transmembrane domains, a large proportion of the protein was found in the membrane fraction. We therefore solubilized this fraction as described previously (Jung, Tjaden & Altendorf 1997) prior to purification. Purification was performed as described before (Heermann, Altendorf, & Jung, 2003), except that 250 mmol/L imidazole was present in the elution buffer.

### 2.12 Analytical procedures

The concentration of soluble proteins was determined as described by Lowry, Rosebrough, Farr, & Randall, (1951) and membrane proteins were quantified with a modified Lowry method (Peterson, 1977) using bovine serum albumin as a standard.

### 2.13 Phosphorylation assay

Purified PhoR (0.2 mg ml^−1^, final concentration) or membrane vesicles containing approximately 0.2 mg ml^−1^ KdpD (final total protein concentration 2 mg ml^−1^), respectively, were incubated in phosphorylation buffer [50 mmol/L Tris/HCl, pH 7.5, 10% glycerol (v/v), 0.5 mol/L NaCl, 10 mmol/L MgCl_2_ and 2 mmol/L dithiothreitol] at room temperature. Phosphorylation was initiated by addition of 20 μmol/L [γ‐^32^P]ATP (2.38 Ci/mmol). At the indicated times, aliquots were removed and the reaction was stopped by mixing with SDS sample buffer (Jung, Tjaden & Altendorf 1997). After incubation for 20.5 min, purified PhoB and KdpE were added at a final concentration of 0.1 mg ml^−1^ to the PhoR‐ and KdpD‐containing samples, respectively (resulting in a 1:2 dilution of PhoR/KdpD, ATP), and the incubation was continued. Aliquots were removed at different times, mixed with SDS sample buffer and subjected to SDS‐PAGE. Gels were then dried and protein phosphorylation was detected by exposure of the gels to a Storage Phosphor Screen. Band intensity was quantified using ImageJ (Schindelin et al., 2015).

## 3 RESULTS

### 3.1 *E. coli* requires the KdpFABC system to grow under K^+^ limitation

**Figure 1:**
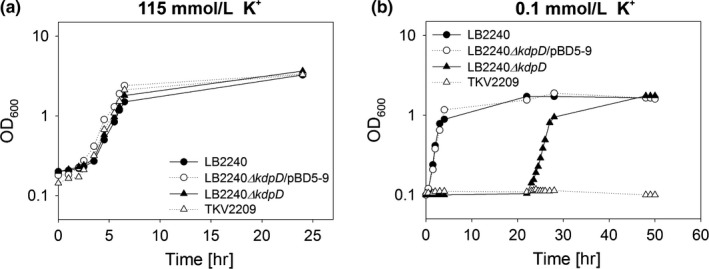
Effects of different K^+^ concentrations on the growth of various *kdp* ^*+/−*^ strains. LB2240 (*kdpD* ^*+*^), LB2240*ΔkdpD*/pBD5‐9 (*kdpD* ^*−*^, complemented with plasmid‐encoded *kdpD*), LB2240*ΔkdpD* (*kdpD* ^*−*^), and TKV2209 (*kdpD* ^*−*^, *kdpE* ^*−*^) were cultivated in minimal medium containing the indicated K^+^ concentrations. (a) Growth of strains in K^+^‐rich medium (115 mmol/L K^+^). Cells were precultivated in medium containing 115 mmol/L K^+^, and inoculated into fresh medium at an initial OD_600_ of 0.1. Growth was monitored for 24 hr. (b) Growth of strains under K^+^ limitation (0.1 mmol/L K^+^). Cells were precultivated in medium containing 115 mmol/L K^+^, washed with K^+^‐free medium and transferred into medium containing 0.1 mmol/L K^+^ at an initial OD_600_ of 0.1. Growth was monitored for 52 hr. The growth curves are representative for at least three biological replicates

In order to determine the role of the histidine kinase KdpD for K^+^‐dependent growth, we generated the *E. coli* strain LB2240*∆kdpD*, which is deleted for *kdpD*, as well as for *trk* and carries a mutated *kup* (*kup* ^*−*^), which encode the two constitutively expressed K^+^ transporters. This strain retains a functional *kdpFABC* operon coding for a high‐affinity uptake system, whose expression is dependent on the phosphorylation of KdpD/KdpE. We tested growth of this strain in K^+^‐limited (0.1 mmol/L K^+^) and K^+^‐rich (115 mmol/L K^+^) medium, and compared the results with those for LB2240 (the parental strain), LB2240*∆kdpD*/pBD5‐9 complemented by a plasmid‐encoded *kdpD* and TKV2209 (which carries an additional deletion in *kdpE)*. All strains were able to grow in K^+^‐rich (115 mmol/L K^+^) medium (Figure 1a). When extracellular K^+^ levels are high, nonspecific uptake is sufficient for growth and no specific transporter is required (Laermann et al., 2013). When these strains were exposed to K^+^ limitation (0.1 mmol/L K^+^), only those carrying either chromosomally (LB2240) or plasmid‐encoded *kdpD* (LB2240*∆kdpD*/pBD5‐9) were able to grow normally (Figure 1b). Strikingly, however, while strain TKV2209 lacking both the *kdpD* and *kdpE* genes was unable to grow under K^+^ limitation, exponential growth of LB2240∆*kdpD* abruptly set in after an initial lag phase of around 22 hr, and ultimately reached the same optical density as the *kdpD* ^*+*^ strains (Figure 1b).

### 3.2 KdpE‐mediated induction of *kdpFABC* expression relieves growth arrest in the absence of KdpD

**Figure 2:**
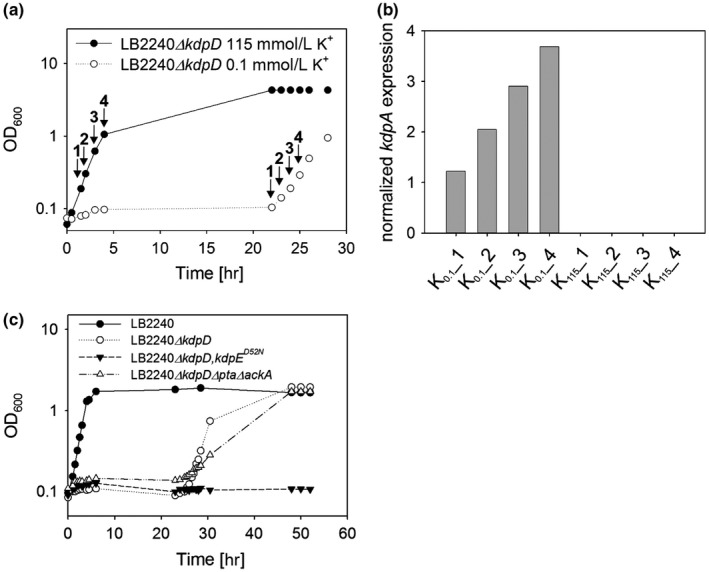
KdpE‐P activates *kdpFABC* expression independently of KdpD and acetyl phosphate. (a) Growth of the indicated mutants in K^+^‐limited (0.1 mmol/L K^+^) and K^+^‐rich (115 mmol/L K^+^) minimal medium. Cells were cultivated as described in Figure 1 and samples were taken at the time points indicated. (b) Samples taken at the time points indicated in **A** were used for qRT‐PCR. RNA was extracted and *kdpA* transcripts were quantified relative to expression of the *gapA* gene. Mean values of three technical replicates are shown, and are representative for biological duplicates. (The standard deviation was >10%). (c) Growth of strains LB2240, LB2240*ΔkdpD*, LB2240*ΔkdpD*,* kdpE* ^*D52N*^, and LB2240*ΔkdpDΔptaΔackA* under K^+^ limitation (0.1 mmol/L K^+^). Cells were cultivated as described in Figure 1b. The growth curves are representative for at least three biological replicates

We hypothesized that KdpE is required to rescue growth of strain LB2240*ΔkdpD* under K^+^‐limiting growth conditions, as TKV2209 cells carrying an additional *kdpE* deletion do not grow in K^+^‐depleted medium. We therefore tested whether KdpE can induce *kdpFABC* expression independently of KdpD. As measure of *kdpFABC* expression, *kdpA* transcripts were quantified by qRT‐PCR in exponentially growing cells of strain LB2240*ΔkdpD* in K^+^‐rich and K^+^‐limited media (Figure 2a, b). As expected, no *kdpA* transcripts were detectable in cells cultivated in medium containing 115 mmol/L K^+^ (Figure 2b). Under K^+^ limitation, we observed a linear increase in *kdpA* transcripts after the 22‐h lag phase (Figure 2b). These results reveal that KdpE can activate *kdpFABC* expression in the absence of KdpD under K^+^‐limiting conditions.

To analyze whether phosphorylation of KdpE is required for the activation of *kdpFABC* transcription, the acceptor site (D52) was inactivated by a chromosomal point mutation that converted the aspartate into an asparagine (D52N). The resulting strain LB2240*ΔkdpD*,*kdpE‐*D52N failed to emerge from growth arrest within 50 hr, whereas the strain LB2240*ΔkdpD*—as described above—started to grow after 22 hr (Figure 2c). Hence, phosphorylation of KdpE is indeed essential for the relief of growth arrest under K^+^ limitation in the absence of KdpD.

We then asked how KdpE can be phosphorylated in the absence of its cognate histidine kinase KdpD. Acetyl phosphate is known to serve as a phosphodonor for KdpE in vitro (Heermann, Altendorf & Jung 2003). In *E. coli* acetyl phosphate is produced as an intermediate of central metabolism, either from the precursors acetyl‐CoA and inorganic phosphate by the phosphotransacetylase Pta or from ATP and acetate by the acetate kinase AckA. In order to test if acetyl phosphate is the phosphodonor for KdpE in LB2240*ΔkdpD*, the genes *pta* and *ackA* were additionally deleted, yielding strain LB2240*ΔkdpDΔptaΔackA*. If acetyl phosphate is responsible for KdpE phosphorylation, this strain should not be able to emerge from growth arrest under K^+^ limitation. On the contrary, we found that it began to grow at around the same time as LB2240*ΔkdpD* under K^+^ limitation (Figure 2c). The growth rate of strain LB2240*ΔkdpDΔptaΔackA* was lower than that of LB2240*∆kdpD* (Figure 2c); however, similar effects were observed when the former was grown in K^+^‐rich medium (data not shown). Therefore, we concluded that acetyl phosphate does not act as a phosphodonor for KdpE in vivo under these conditions.

### 3.3 Only a very small subpopulation of strain LB2240Δ*kdpD* survives K^+^ limitation

**Figure 3:**
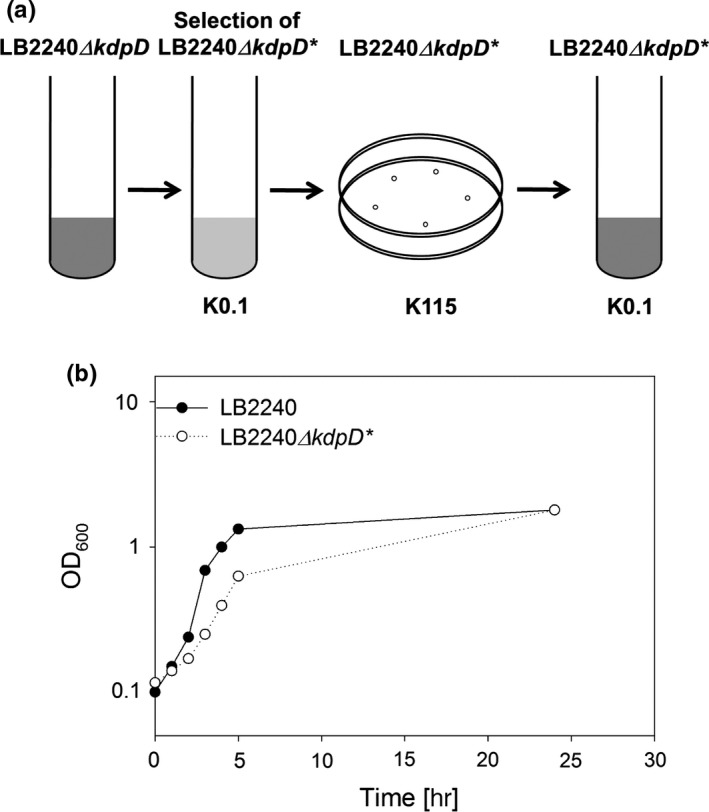
Isolation and growth of LB2240ΔkdpD* under K^+^ limitation. (a) Schematic depiction of the procedure used to isolate LB2240ΔkdpD* mutants. (b) *E. coli* strain LB2240*ΔkdpD* was grown in medium containing 115 mmol/L K^+^, inoculated into K^+^‐limited medium (0.1 mmol/L K^+^) and grown to stationary phase. Dilutions were then spread on plates containing 115 mmol/L K^+^. Single clones were inoculated into liquid K^+^‐limited medium, and growth was monitored over time and compared with that of strain LB2240

Next, we wanted to know whether the whole population of LB2240*ΔkdpD* cells is able to adapt to K^+^ limitation, or if only a subpopulation finds a way to induce *kdpFABC* expression in the absence of KdpD. LB2240*ΔkdpD* was cultivated as described before in minimal medium containing 115 mmol/L K^+^. Then about 10^8^ cells were spread on plates with minimal medium containing 0.1 mmol/L K^+^, and incubated at 37°C. On average, five colonies grew from 10^8^ cells on the K^+^‐limited plates, whereas cells of strain LB2240 grew as a bacterial lawn (data not shown). This result provided the first hint that suppressor mutations were being generated in strain LB2240*ΔkdpD* during the long lag phase. If so, the isolated clones should grow under K^+^ limitation without an extended lag phase. To test this prediction, we inoculated the parental strain in K^+^‐limited medium and plated the outgrowing cells on agar plates containing 115 mmol/L K^+^. Afterward, single colonies were inoculated into K^+^‐limited liquid minimal medium and growth was monitored over time (Figure 3a). As expected, these single clones (from now on called LB2240ΔkdpD*) were able to grow under K^+^ limitation without an extended lag phase (Figure 3b).

### 3.4 Mutations in the phosphate transporter PstCAB result in *kdpFABC* expression

We then set out to identify the suppressor mutation that enables *kdpFABC* to be expressed in the absence of KdpD in LB2240ΔkdpD* strains. To do so, we performed whole‐genome shotgun sequencing of several clones of LB2240ΔkdpD* on the Illumina platform. By comparing these sequences to the reference genome of LB2240*ΔkdpD*, we detected a single base deletion in the *pstC* gene, which resulted in a shift of the open reading frame. In two other clones we found mutations in the gene coding for the 257‐aa PstB protein, resulting in a stop codon (S_50_Stop) and a shift in the open reading frame after 86 codons, respectively. *pstC* and *pstB* form part of the *pstSCAB* operon, which codes for the high‐affinity phosphate transporter PstCAB, together with the periplasmic phosphate‐binding protein PstS (Amemura, Makino, Shinagawa, Kobayashi, & Nakata, 1985; Rees, Johnson, & Lewinson, 2009; Webb, Rosenberg, & Cox, 1992). The PstCAB transporter is known to act as a phosphate sensor for the two‐component system PhoR/PhoB and forms a signaling complex together with the PhoU protein (Gardner et al., 2014). Deletion of *phoU* or any of the transporter genes *pstCAB* shifts the histidine kinase PhoR into the constitutive kinase “ON” state.

**Figure 4:**
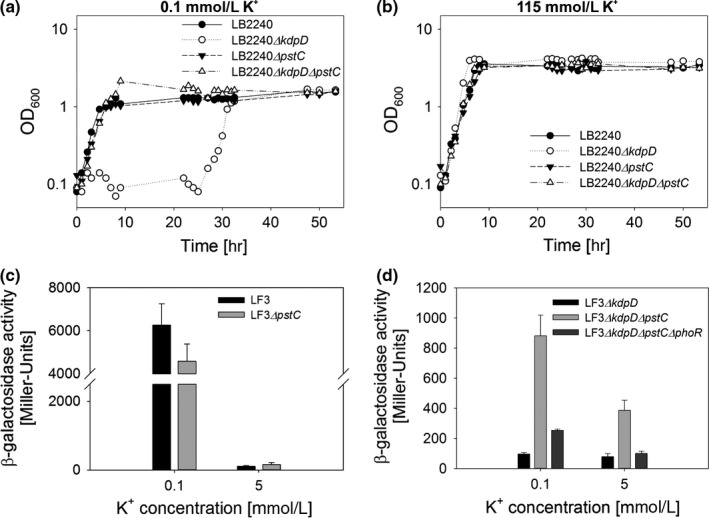
*pstC* deletion mutants induce *kdpFABC* in the absence of KdpD. (a, b) Growth curves of strains LB2240, LB2240*ΔkdpD*, LB2240*ΔpstC,* and LB2240*ΔkdpDΔpstC* in K^+^‐limited (a) and K^+^‐rich minimal medium (b). Cells were cultivated as described in Figure 1 and growth was monitored for 52 hr. Graphs are representative for two biological replicates. (c, d) β‐Galactosidase activities of the reporter strains LF3 (c), LF3*ΔpstC* (c), LF3*ΔkdpD* (d), LF3*ΔkdpDΔpstC* (d), and LF3*ΔkdpDΔpstCΔphoR* (d). In all strains the native *lacZ* promoter region was replaced by the *kdpFABC* promoter region (chromosomal *P*_*kdpFABC*_ *::lacZ* fusion) and β‐galactosidase activities were determined after cultivation of cells in minimal medium containing the indicated concentrations of K^+^. The plots show means and standard deviations for at least three biological replicates

In order to verify the sequencing results, we deleted *pstC* in LB2240*ΔkdpD* and tested for growth of the resulting mutant under K^+^ limitation (Figure 4a). Indeed, we observed that, under K^+^ limitation, the strain carrying the double deletion in *kdpD* and *pstC* (LB2240*ΔkdpDΔpstC*) resumed growth with no lag phase directly after inoculation, whereas the single *kdpD* deletion led to a growth arrest as described above (Figures 4a, 1b). All strains grew well in K^+^‐rich medium (Figure 4b).

To confirm that a deletion in *pstC* would result in *kdpFABC* expression we performed reporter gene assays with strain LF3, in which the *kdpFABC* promoter is fused to the *lacZ* gene at the native *lacZ* gene locus (Fried, Lassak, & Jung, 2012) and the endogenous *kdpFABC* and *kdpDE* operons as well as the two constitutively expressed *trk* and *kup* K^+^ transporter genes are intact. In LF3, *kdpFABC* expression—measured indirectly via β‐galactosidase activity—is induced if the extracellular K^+^ concentration falls below 5 mmol/L (Figure 4c) (Fried, Lassak & Jung 2012). A deletion in *pstC* did not affect the *kdpFABC* expression pattern (Figure 4c). However, if the reporter strain carries an additional deletion in the *kdpD* gene (LF3*ΔkdpDΔpstC*) strong induction of *kdpFABC* was found at 5 mmol/L K^+^, and was further increased at 0.1 mmol/L K^+^ (Figure 4d). As expected, the *kdpD* deletion mutant (LF3*ΔkdpD*) was unable to express *kdpFABC* (Figure 4d).

Taken together, these results show that disabling point mutations or deletions in one or other of the *pst* transporter components rescues growth defects under K^+^ limitation by inducing *kdpFABC* expression in the absence of KdpD.

### 3.5 The histidine kinase PhoR is responsible for *kdpFABC* expression in the absence of KdpD

**Figure 5:**
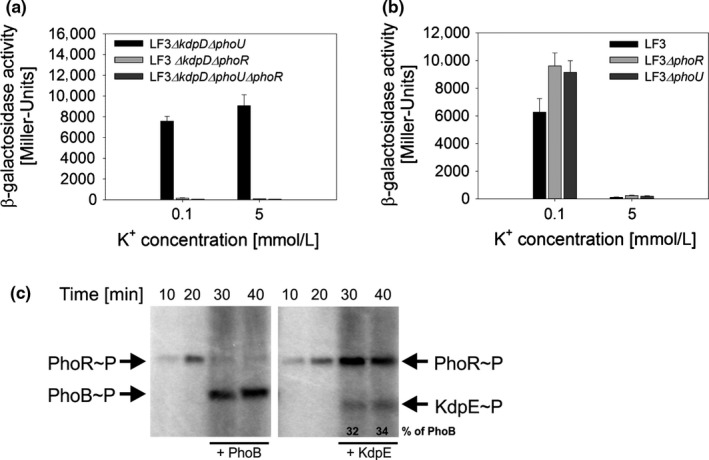
The histidine kinase PhoR can phosphorylate KdpE. (a , b) β‐Galactosidase activities of the reporter strains LF3 and corresponding deletion mutants. In all strains the native *lacZ* promoter region was replaced by the *kdpFABC* promoter region (chromosomal *P*_*kdpFABC*_ *::lacZ* fusion) and β‐galactosidase activities were determined after growth of cells in minimal medium at the indicated K^+^ concentrations. The histograms depict means and standard deviations for at least three biological replicates. (c) In vitro autophosphorylation of PhoR with [γ‐^32^P]ATP (time points 10 and 20 min). After 20.5 min, PhoB or KdpE was added and phosphotransfer was monitored. Phosphorylated proteins were subjected to SDS‐PAGE and gels were exposed to a phosphoscreen. Each autoradiograph is representative for two independent experiments. Band intensity of phosphorylated partner and nonpartner response regulators were quantified and are indicated in percent

Signal perception by the histidine kinase PhoR occurs via interaction with the phosphate transporter PstCAB and the negative regulator PhoU (Gardner et al., 2014; Hsieh & Wanner, 2010; Lamarche et al., 2008). As a deletion in *phoU* or *pstCAB* shifts PhoR into the kinase “ON” state, we inferred that it could act as a phosphodonor for KdpE in the absence of KdpD. To test this possibility, we deleted *phoR* in the reporter strain LF3 lacking *kdpD* and *pstC* (resulting in strain LF3*ΔkdpDΔpstCΔphoR*) and quantified *kdpFABC* expression via β‐galactosidase activity after cultivating the cells in minimal medium containing different K^+^ concentrations. As expected, we observed no *kdpFABC* expression in the double deletion mutant LF3*ΔkdpDΔphoR* or in the triple mutant LF3*ΔkdpDΔpstCΔphoR*, which supports the idea that PhoR serves as phosphodonor for KdpE (Figures 4d, 5a).

Subsequently, we tested the effect of the accessory protein PhoU on the Kdp system. The reporter strain LF3*ΔkdpD* carrying a deletion in *phoU* (LF3*ΔkdpDΔphoU*) showed high induction of *kdpFABC* expression independently of the extracellular K^+^ concentration. Here again, functional deletion of *phoR* (strain LF3*ΔkdpDΔphoUΔphoR*) prevented induction (Figure 5a). Note that induction of *kdpFABC* expression was around 10‐fold higher in the *phoU* deletion mutant (LF3*ΔkdpDΔphoU*) than in the *pstC* deletion mutant (LF3*ΔkdpDΔpstC*) (cf. Figures 5a and 4d). However, *phoR* and *phoU* deletion mutants harboring an intact KdpD induced *kdpFABC* expression comparable to the parental strain LF3 (Figure 5b).

To further verify that PhoR acts as phosphodonor for KdpE we assayed phosphotransfer from PhoR to its cognate response regulator PhoB and to the noncognate KdpE. A truncated PhoR lacking the transmembrane domains (Lüttmann, Göpel, & Görke, 2012) was autophosphorylated in the presence of [γ‐^32^P]ATP. After 20.5 min the response regulators PhoB and KdpE, respectively, were added and phosphotransfer was monitored over time. We found that PhoR phosphorylates not only PhoB, but also KdpE—albeit to a lesser extent (Figure 5c). Taking all these data together, we conclude that PhoR is responsible for phosphorylation of KdpE in the absence of KdpD.

### 3.6 Bacterial adenylate cyclase two‐hybrid experiments indicate in vivo interactions between the two‐component systems KdpD/KdpE and PhoR/PhoB

**Figure 6:**
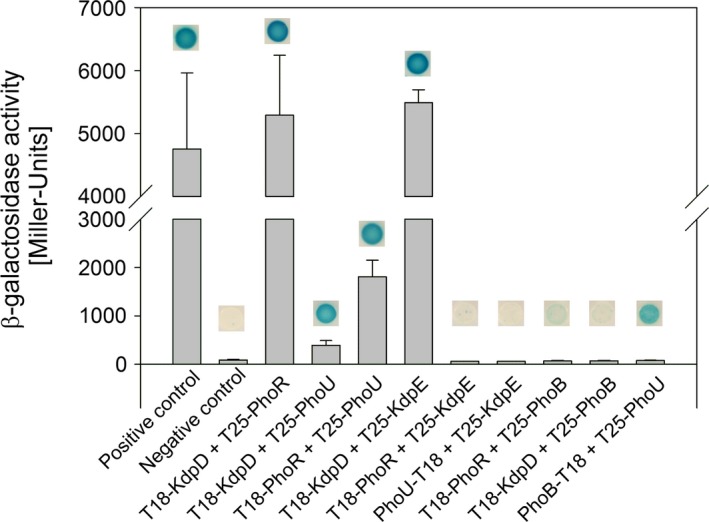
Bacterial adenylate cyclase two‐hybrid experiments indicate interactions between the two‐component systems KdpD/KdpE and PhoR/PhoB. Fragments T18 and T25 of *Bordetella pertussis* CyaA were fused to proteins of interest as indicated. The hybrids with yeast leucine zipper fragments were used as a positive control and the fragments T18 and T25 alone as negative control. *E. coli* BTH101 was cotransformed with plasmid pairs coding for indicated hybrid proteins and cultivated under aerobic conditions. The prefixes T18 and T25 indicate fragments of CyaA N‐terminally fused to the protein of interest, the suffixes refer to C‐terminal T18 or T25 fusions. For plate assays cells were cultivated in LB medium overnight, washed and subsequently spotted on LB plates. All plates were supplemented with ampicillin, kanamycin, IPTG, and X‐Gal as described in Experimental Procedures and were incubated at 25°C for 72 hr. For quantification of β‐galactosidase activity cells were cultivated in LB medium supplemented with ampicillin, kanamycin and IPTG as described in Experimental Procedures at 25°C for 48 hr. The activity of the reporter enzyme β‐galactosidase was determined and served as a measure of the interaction strength. The histograms show means and standard deviations for at least three biological replicates

To determine whether components of the two signaling systems interact with each other in vivo, we made use of the bacterial adenylate cyclase two‐hybrid system (BACTH). The leucine‐zipper fusion constructs zip‐T18 and T25‐zip from the yeast *Saccharomyces cerevisiae* were used as positive control and the proteins T18 and T25 alone as negative controls. In the first screen we tested for interactions on LB plates. (Figure 6). The hybrid protein T18‐KdpD was found to interact strongly with T25‐KdpE, T25‐PhoR, and T25‐PhoU on LB plates (Figure 6). There was no detectable interaction between T18‐KdpD and the noncognate response regulator hybrid T25‐PhoB. For T18‐PhoR the assay revealed interaction with T25‐PhoU, but not with T25‐PhoB or T25‐KdpE. Furthermore, the BACTH test indicated interactions between PhoB‐T18 and T25‐PhoU, but not between PhoU‐T18 and T25‐KdpE. Note that we constructed hybrids in all possible combinations, and all cases that yielded negative results were confirmed with the opposite cloning permutation (data not shown). To quantify the strengths of the interactions we determined β‐galactosidase activity after culturing cells in liquid medium (Figure 6). As expected, high β‐galactosidase activities were detected in cells producing T18‐KdpD+T25‐KdpE and T18‐KdpD+T25‐PhoR, respectively, and moderate to low activities were measured in cells producing T18‐KdpD+T25‐PhoU and T18‐PhoR+T25‐PhoU. No activity was detectable for any other combination. The only discrepancy we observed concerns the PhoB‐T18/T25‐PhoU pair, for which interaction was signaled when cells were grown on plates, but not in liquid culture (Figure 6). This result implies that the interaction is very weak and becomes detectable by this assay only after long incubation times and persistent accumulation of β‐galactosidase protein. Finally, we did not find any effect of the external K^+^ and ${\text{PO}}_{4}^{3-}$ concentrations on the interaction strengths of the tested constructs (data not shown).

In summary, the BACTH assay indicated that KdpD and PhoR, KdpD and PhoU, and PhoR and PhoU can interact with each other, respectively. These data support the assumption that both PhoR and PhoU can interact with KdpD and influence its activity. In this assay we did not detect interaction of PhoR with PhoB or KdpE, which might be explained by steric hindrance due to the fused adenylate cyclase, or wrong orientation of the two halves of the adenylate cyclase. Furthermore, it might be that the interaction between PhoR and the histidine kinase is only transient as it was shown before for other histidine kinase/response regulator pairs (Zapf, Sen, Madhusudan, Hoch, & Varughese, 2000).

### 3.7 Phosphate limitation enhances *kdpFABC* expression

**Figure 7:**
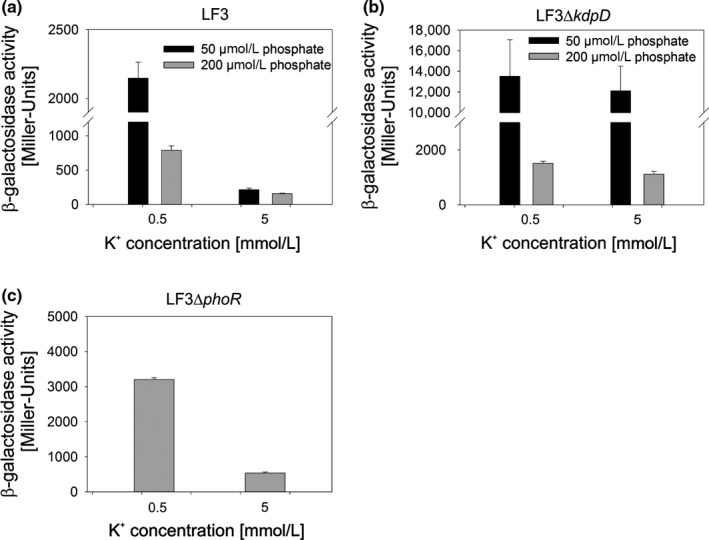
Phosphate limitation enhances *kdpFABC* expression. (a, b) β‐Galactosidase activities of the reporter strains LF3 (a), LF3*ΔkdpD* (b) and LF3*ΔphoR* (c). In all strains the native *lacZ* promoter region was replaced by the *kdpFABC* promoter region (chromosomal *P*_*kdpFABC*_ *::lacZ* fusion) and β‐galactosidase activities were determined after cultivation of cells in Tris‐maleic acid (TMA) minimal medium at the indicated K^+^ and ${\text{PO}}_{4}^{3-}$ concentrations. For LF3*ΔphoR* 50 μmol/L phosphate was used. The histograms show means and standard deviations for at least three biological replicates

Thus far, our results indicate cross‐talk between KdpE and PhoR/PhoB in the absence of KdpD. But can it occur in an intact system? If so, it would permit functional coupling of K^+^ and ${\text{PO}}_{4}^{3-}$ homeostasis. To answer this question, we cultivated the reporter strain LF3 in defined TMA medium supplemented with different concentration of K^+^ and ${\text{PO}}_{4}^{3-}$. As shown before (Fried, Lassak & Jung, 2012) there is basically no induction of *kdpFABC* in K^+^‐rich medium (5 mmol/L) (Figure 7a). Under moderate K^+^ limitation (0.5 mmol/L K^+^) *kdpFABC* expression is induced via KdpD. Notably, we found a threefold higher induction when cells were simultaneously exposed to K^+^ and phosphate limitation (50 μmol/L phosphate) (Figure 7a). The *kdpD* deletion mutant (LF3*ΔkdpD*) exhibited K^+^‐independent *kdpFABC* expression, which was also enhanced under phosphate limitation (Figure 7b). However, the observed upregulation was probably not solely dependent on the histidine kinase PhoR as indicated by studies with mutant LF3*ΔphoR* (Figure 7c). It should be noted, that overall *kdpFABC* expression was found to be higher in the *phoR* mutant than in the PhoR^+^ strain (see also Figure 5b). We conclude that *kdpFABC* expression is fine‐tuned under simultaneous phosphate limitation, which indicates cross‐regulation between these two systems.

### 3.8 K^+^ limitation enhances expression of *pstS*

**Figure 8:**
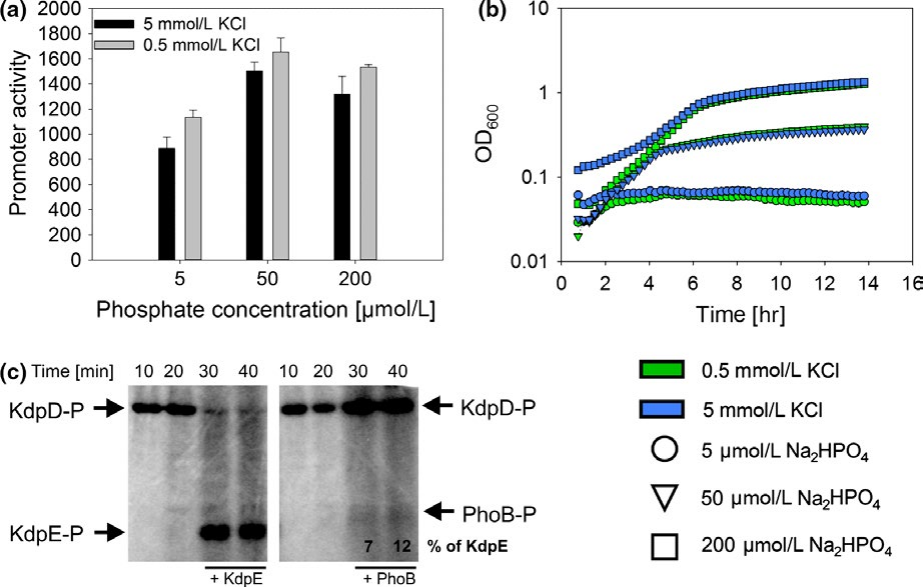
K^+^ limitation enhances *pstS* expression, and PhoB phosphorylation by KdpD. (a**)** *pstS* promoter activity in *E. coli* MG1655 cells carrying plasmids in which *mcherry* expression is under the control of the *pstS* promoter. Cells were cultivated in Tris‐maleic acid (TMA) minimal medium containing the indicated K^+^ and ${\text{PO}}_{4}^{3-}$ concentrations. Shown is the maximal promoter activity, which was observed after 2 hr at 5 μmol/L Na_2_HPO_4_, 5 hr at 50 μmol/L Na_2_HPO_4_, and after 7.5 hr at 200 μmol/L Na_2_HPO_4_. Shown is the mean and standard deviation of three independent experiments. (b) Corresponding growth curves of strains cultivated in Tris‐maleic acid (TMA) minimal medium containing different K^+^ and ${\text{PO}}_{4}^{3-}$ concentrations as indicated in green and blue symbols refering to K^+^‐limited (0.5 mmol/L) and K^+^‐rich (5 mmol/L) medium, respectively. Circles, triangles, and squares depict the indicated Na_2_HPO_4_ concentration. Shown is the mean and standard deviation of three independent experiments. (c) In vitro autophosphorylation of KdpD with [γ‐^32^P]ATP (time points 10 and 20 min). After 20.5 min PhoB or KdpE was added and phosphotransfer was monitored. Phosphorylated proteins were subjected to SDS‐PAGE and gels were exposed to a phosphoscreen. Each autoradiograph is representative for two independent experiments. Band intensity of phosphorylated partner and nonpartner response regulators were quantified and are indicated in percent

Having shown that phosphate limitation has an impact on *kdpFABC* expression, we asked whether K^+^ limitation reciprocally affects the PhoR/PhoB system, which regulates expression of the *pho* regulon comprising more than 30 target genes, including the *pstSCAB* operon. To analyze PhoB activity we used *E. coli* MG1655 cells transformed with a plasmid‐based reporter system, in which the *pstS* promoter is fused to *mcherry*. Cells were cultivated in the defined TMA medium containing different levels of K^+^ and ${\text{PO}}_{4}^{3-}$, and growth and fluorescence were monitored over time. Promoter activity was quantified by computing the increase in fluorescence intensity per unit time relative to the optical density of the culture (Bren et al., 2013). We observed an early activation of the *pstS* promoter under extreme phosphate limitation (5 μmol/L phosphate, maximal induction after 2 hr growth) and delayed activation under moderate ${\text{PO}}_{4}^{3-}$ limitation (50 μmol/L and 200 μmol/L phosphate, maximal induction after 5 and 7.5 hr of growth, respectively) (Figure 8a). Notably, *pstS* promoter activity exhibited a slight additional increase when cells were simultaneously exposed to K^+^ limitation, indicating cross‐phosphorylation from KdpD to PhoB (Figure 8a). Bacterial growth was clearly determined by phosphate availability, and not by the external K^+^ concentration (Figure 8b).

To confirm cross‐regulation from KdpD to PhoB we tested autophosphorylation of KdpD in membrane vesicles and phosphotransfer in vitro. We observed phosphotransfer from KdpD‐P not only to KdpE, but to PhoB as well, albeit to a comparably minor extent (Figure 8c). These results corroborate the idea of cross‐regulation between the two signal transduction systems to fine‐tune the response depending on K^+^ and phosphate availability.

## 4 DISCUSSION

Bacteria predominantly use two‐component signal transduction to adapt to changing environmental conditions (Stock, Robinson, & Goudreau, 2000). A prototypical two‐component system consists of a membrane‐integrated sensor kinase and a cytoplasmic response regulator that mediates the cellular response. *E. coli* has at least 30 two‐component systems that monitor and respond to an array of environmental and cellular parameters including temperature, extracellular pH and osmolarity, and constituents such as essential nutrients. A variety of receptor domains and their corresponding triggers have been identified and underline the importance of the cell's ability to sense and adapt to fluctuating conditions (for review, see (Mascher, Helmann, & Unden, 2006; Krell et al., 2010; Szurmant, White, & Hoch, 2007). However, how specificity is maintained between these signal transduction systems is not fully understood. Podgornaia & Laub (2013) suggested three key mechanisms that could serve to define the specificity of individual two‐component signal transduction systems: molecular recognition, phosphatase activity, and substrate competition. According to these authors, the dominant basis for specificity mechanism is molecular recognition, that is the strong kinetic preference of a histidine kinase for its partner response regulator in vitro (Skerker, Prasol, Perchuk, Biondi, & Laub, 2005). Most histidine kinases are bifunctional enzymes, having both kinase and phosphatase activities (Willett & Kirby, 2012). The phosphatase activity is assumed to counteract unspecific phosphorylation by noncognate histidine kinases in vivo (Alves & Savageau, 2003). Although cross‐talk between two‐component systems has been described several times, it mainly occurs in the absence of either the cognate histidine kinase or response regulator (Fisher, Jiang, Wanner, & Walsh, 1995; Haldimann, Fisher, Daniels, Walsh, & Wanner, 1997; Silva, Haldimann, Prahalad, Walsh, & Wanner, 1998; Siryaporn & Goulian, 2008). Therefore, it is still not clear whether cross‐talk between two‐component systems is a widespread, but basically incidental phenomenon, or might also be of physiological importance in vivo.

In this study, we demonstrate cross‐regulation between the KdpD/KdpE and PhoR/PhoB two‐component systems of *E. coli*. The KdpD/KdpE system regulates expression of the high‐affinity K^+^ uptake system KdpFABC. We found that, while a mutant lacking KdpD and the two constitutively produced low‐affinity K^+^ uptake systems Trk and Kup are in principle unable to grow under K^+^ limitation, a subpopulation emerges after a 22‐hr lag phase, which subsequently expands at a virtually wild‐type rate (Figure 1). Recovery of growth in these cells was dependent on a phosphorylatable KdpE and induction of *kdpFABC* expression. Whole‐genome sequencing revealed suppressor mutations in the *pstC* and *pstB* genes and further experiments confirmed that a deletion in *pstC* or *phoU*—all of which are supposed to switch the histidine kinase PhoR into the constitutive kinase “ON” state—induced *kdpFABC* expression in the absence of KdpD. These observations provide compelling evidence for cross‐regulation between these two systems. It has been shown previously that, in the absence of PhoR, the response regulator PhoB can be activated by noncognate histidine kinases, including KdpD (Zhou et al., 2005). In addition, an indirect link between these two‐component systems has been described recently: Nonphosphorylated enzyme IIA of the Ntr‐PTS system in *E. coli* has been shown to interact with both KdpD and PhoR, and to stimulate their activities, coupling carbon metabolism with K^+^ and phosphate homeostasis, respectively (Lüttmann et al., 2009).

**Figure 9:**
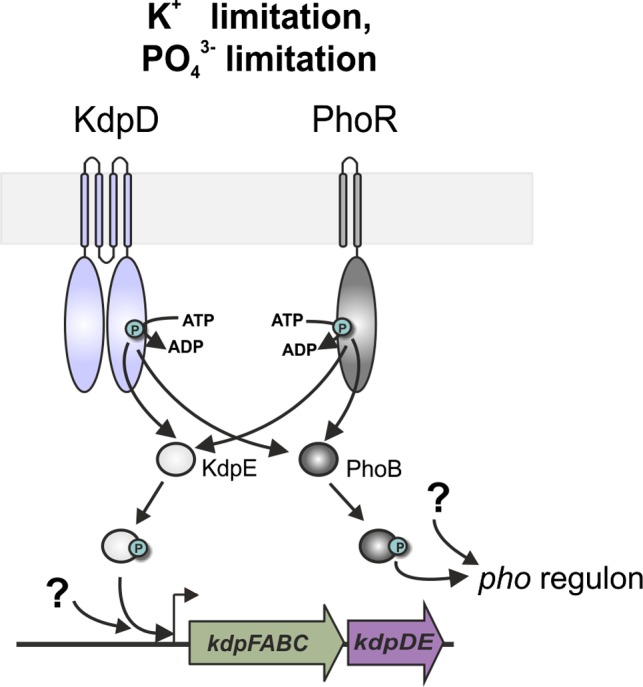
Cross‐regulation of the *kdpFABC* operon and the *pho* regulon couples K^+^ to ${\text{PO}}_{4}^{3-}$ homeostasis in *E. coli*. KdpD and PhoR phosphorylate their cognate response regulators KdpE and PhoB, respectively. Under simultaneous K^+^ and phosphate limitation an additional increase in target gene expression can be achieved by direct cross‐regulation between the two‐component systems and so far unknown regulatory components

Our results thus reveal bidirectional cross‐talk between the *kdpFABC* and *pstSCAB* operons. We found that both histidine kinases can reciprocally phosphorylate the corresponding noncognate response regulator in vitro, and we detected fine‐tuned cross‐regulation of their target genes in vivo. We therefore propose that PhoR activates KdpE and KdpD activates PhoB when cells are simultaneously exposed to both K^+^ and phosphate limitation (Figure 9). Under phosphate limitation, PhoR autophosphorylates and transfers the phosphoryl group to PhoB, which in turn activates transcription of the *pho* regulon. Moreover, PhoR also phosphorylates KdpE. However, as intra‐ and extracellular K^+^ levels are sufficient, the counteracting phosphatase activity of KdpD ensures that KdpE is dephosphorylated and thus prevents *kdpFABC* expression. Under simultaneous K^+^ limitation, however, KdpD is in the kinase active state and phosphorylates KdpE as well as PhoB, thereby boosting K^+^ uptake and transcription of the *pho* regulon. In addition, our BACTH results suggest interactions between KdpD and PhoR, as well as between KdpD and PhoU. Formation of heterodimers between KdpD and PhoR is an attractive, but as yet untested, hypothesis that could account for these results. Besides direct cross‐phosphorylation between these two two‐component systems, our results also suggest that other still unknown components might play a role in the cross‐regulation of these operons. While the increased *kdpFABC* expression in strains lacking *kdpD* was clearly determined by PhoR (Figure 4d and 5a), PhoR had a minor influence in KdpD^+^ cells (Figure 5b). Moreover, cross‐regulation of *kdpFABC* expression under concurrent phosphate and K^+^ limitation was probably not solely dependent on PhoR (Figure 7c). Therefore, it remains still unclear whether interactions between PhoR, KdpD, and PhoU influence the phosphotransfer to the response regulators or whether other regulatory components—unrelated to the Kdp and Pho two‐component systems—are responsible for regulating expression of the *kdpFABC* operon and the *pho* regulon under these stress conditions.

In 1967, it was reported that K^+^ is important for ${\text{PO}}_{4}^{3-}$ uptake (Weiden et al., 1967), but the molecular basis for this phenomenon remained unclear. The cross‐connections between the two uptake systems described here not only provide a possible explanation, but uncover an elegant cellular mechanism for fine‐tuning the ratio of positively and negatively charged ions in the cytoplasm. It should be noted that during the preparation of this manuscript Moreau & Loiseau (2016) published a study about suppressor mutants generated under phosphate starvation. Interestingly one of the mutations the authors identified was located in the *kdpD* gene and resulted in a constitutively active KdpD protein. According to our model, a constitutively active KdpD protein rescues growth of the mutant under phosphate starvation by directly activating PhoB.

In summary, we demonstrate that cross‐regulation between the *kdpFABC* and *pstSCAB* operons occurs under conditions of K^+^ and phosphate limitation. This cross‐regulation interconnects K^+^ and phosphate homeostasis in *E. coli* and fine‐tunes the ratio of positively and negatively charged ions within cells.

## CONFLICT OF INTEREST

None declared
